# A Survey of the State of the Art in Monocular 3D Human Pose Estimation: Methods, Benchmarks, and Challenges

**DOI:** 10.3390/s25082409

**Published:** 2025-04-10

**Authors:** Yan Guo, Tianhan Gao, Aoshuang Dong, Xinbei Jiang, Zichen Zhu, Fuxin Wang

**Affiliations:** Software College, Northeastern University, Shenyang 110004, China; guoyan@stumail.neu.edu.cn (Y.G.);

**Keywords:** pose estimation survey, 3D human pose estimation, deep learning-based pose estimation, single- and multi-person pose estimation

## Abstract

Three-dimensional human pose estimation (3D HPE) from monocular RGB cameras is a fundamental yet challenging task in computer vision, forming the basis of a wide range of applications such as action recognition, metaverse, self-driving, and healthcare. Recent advances in deep learning have significantly propelled the field, particularly with the incorporation of state-space models (SSMs) and diffusion models. However, systematic reviews that comprehensively cover these emerging techniques remain limited. This survey contributes to the literature by providing the first comprehensive analysis of recent innovative approaches, featuring diffusion models and SSMs within 3D HPE. It categorizes and analyzes various techniques, highlighting their strengths, limitations, and notable innovations. Additionally, it provides a detailed overview of commonly employed datasets and evaluation metrics. Furthermore, this survey offers an in-depth discussion on key challenges, particularly depth ambiguity and occlusion issues arising from single-view setups, thoroughly reviewing effective solutions proposed in recent studies. Finally, current applications and promising avenues for future research are highlighted to guide and inspire ongoing innovation in the area, with emerging trends such as integrating large language models (LLMs) to provide semantic priors and prompt-based supervision for improved 3D pose estimation.

## 1. Introduction

Human pose estimation (HPE) is a long-standing problem in computer vision that focuses on determining the spatial locations of key human body joints from sensor data, especially images and videos. Recent breakthroughs in deep learning have significantly advanced HPE, enabling robust performance in real-world applications such as self-driving, virtual try-on, action recognition, and metaverse. While 2D human pose estimation provides valuable information by detecting joint positions on a plane, 3D human pose estimation takes this a step further by reconstructing the depth of the scene. This additional dimension enables a more accurate and detailed understanding of human movement, enhancing applications that require precise spatial analysis. However, this improvement also introduces additional challenges, such as handling depth ambiguities and necessitating more complex data collection processes and sophisticated modeling techniques.

To tackle 3D HPE, various sensing modalities have been explored, each with distinct advantages and limitations. Multi-view methods [1,2] exploit triangulation and geometric consistency across several camera perspectives, considerably enhancing estimation accuracy but at the expense of complex setups and precise calibration, limiting their applicability in large-scale or uncontrolled environments. Sensor-based techniques [3,4,5,6], such as depth cameras or inertial measurement units (IMUs), offer direct and robust 3D measurements resistant to visual occlusion. However, these approaches involve higher hardware costs, specialized or wearable equipment, potential drift issues, and reduced user comfort with extended usage. In contrast, monocular RGB methods, which infer 3D pose from single-view 2D images, have attracted substantial attention due to their low cost, ease of deployment, and scalability. Despite their inherent reliance on learned priors and the challenging nature of accurately reconstructing depth, these methods offer lightweight, non-intrusive solutions adaptable to real-world, unconstrained scenarios. Consequently, this survey emphasizes monocular RGB-based 3D HPE, highlighting its practical significance and broad applicability.

In earlier research, traditional approaches for HPE employed handcrafted feature descriptors like Histogram of Oriented Gradients (HOG) [7] and Scale-Invariant Feature Transform (SIFT) [8]; combined with heuristic spatial models, these were effective in controlled scenarios but struggled with adaptability and complexity in realistic, dynamic environments. The advent of deep learning methods, particularly convolutional neural networks (CNNs), significantly addressed these limitations through automatic feature extraction and complex nonlinear modeling, substantially improving both accuracy and robustness, and laying the groundwork for subsequent breakthroughs in 3D human pose estimation. Subsequent approaches have increasingly incorporated structural insights into the estimation process. Graph convolutional networks (GCNs) were introduced to explicitly represent the human skeleton by modeling interdependencies among joints and enforcing anatomical constraints, thereby enhancing robustness to occlusion. Additionally, temporal convolutional networks (TCNs) were employed to capture long-range temporal dependencies in sequences, resulting in smoother, temporally consistent pose predictions. More recently, the field has witnessed the emergence of transformer-based architectures. For example, Zheng et al. [9] utilize spatio-temporal self-attention mechanisms to model both spatial and temporal features in video data, pushing the state of the art on various benchmarks. Further advances include generative diffusion models to handle inherent ambiguities by producing multiple plausible pose hypotheses, and the adoption of state-space models (SSMs) for efficient long-sequence modeling with reduced computational overhead. Collectively, these innovations have transformed 3D human pose estimation into a sophisticated and versatile framework capable of robust performance in real-world scenarios.

Several surveys, as summarized in Table 1, have documented the evolution of human pose estimation, with earlier works [10,11,12] predominantly addressing traditional approaches and subsequent studies [13,14,15] emphasizing deep learning methodologies. Despite these extensive efforts, there remains a significant gap in the literature. Recently introduced innovative techniques, such as SSMs and diffusion models, are reshaping the field, offering novel approaches to address challenges like depth ambiguity and occlusion. These innovations, however, have not been thoroughly examined in existing surveys. Consequently, an updated review is required, revisiting established deep learning strategies while comprehensively analyzing emerging paradigms, including diffusion-based and mamba-based methods. The literature is categorized according to input modality (RGB images versus videos) and application scenario (single-person versus multi-person estimation).

The main contributions and highlights of this survey are as follows.

- A comprehensive review of contemporary deep learning-based techniques for 3D human pose estimation is provided, including systematic analysis of emerging innovations such as diffusion models and state-space models within the 3D HPE domain.
- 3D HPE methods from monocular RGB data are systematically categorized based on their input modalities and architectural designs, with an emphasis on their distinctive features, advantages, and limitations.
- A critical analysis of the challenges inherent to monocular 3D HPE—such as dataset limitations, depth ambiguity, occlusion, and motion artifacts—is provided, along with a discussion of potential solutions.
- Emerging research avenues are explored, including data-efficient learning, cross-domain generalization, real-time performance, and the integration of novel paradigms such as large language models (LLMs), neural radiance fields (NeRFs), and physics-based constraints, thereby providing valuable guidance for advancing the state of monocular 3D HPE.

**Figure 1:**
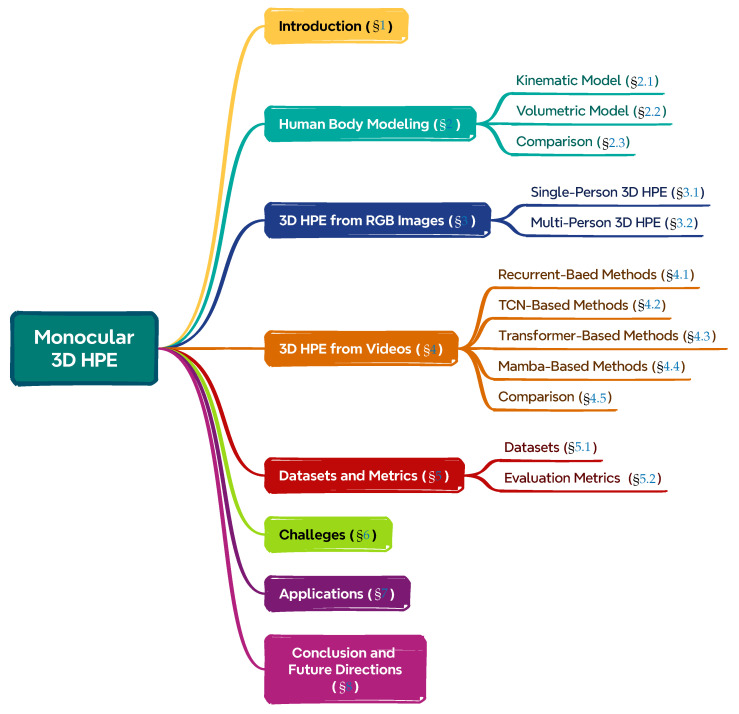
A taxonomy of the survey.

A summary of the structure of this article can be found in Figure 1. The remainder of this survey is structured as follows. Section 2 reviews human modeling techniques relevant to HPE. Section 3 and Section 4 provide detailed discussions on 3D HPE from RGB images and videos, respectively. Section 5 summarizes the commonly used datasets and evaluation metrics, while Section 6 delves into the existing challenges. Section 7 explores diverse application domains, and finally, Section 8 concludes the paper with insights on future research directions.

## 2. Human Body Models

The human body model is an essential component of HPE since it represents the keypoints and features of the human body extracted from the input. Since the human body is flexible and complicated, the human body model must be capable of adapting to a wide range of poses and movements, rather than merely static postures. Furthermore, considering the variation in height, body shape, and proportions across individuals, the model must be adaptable to suit the diversity and morphological changes of various human bodies. Currently, there are two primary forms of human models in 3D HPE, which are the kinematic model and the volumetric model.

### 2.1. Kinematic Model

**Figure 2:**
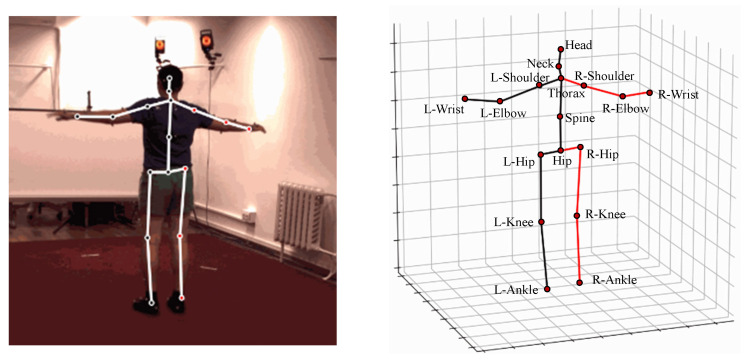
The annotations of each joint in the Human3.6M dataset.

The kinematic model directly abstracts the human body as line segments, with each joint representing a specific body part and connected based on inherent skeletal relationships. The skeletal structure of the human body is displayed in a 3D coordinate system to describe the human posture. Three-dimensional human pose datasets vary in the number of joints they include; for instance, Human3.6M [21] features 17 joints, while HumanEva [22] has 15. The annotations of each joint in the Human3.6M dataset are shown in Figure 2.

### 2.2. Volumetric Model

**Figure 3:**
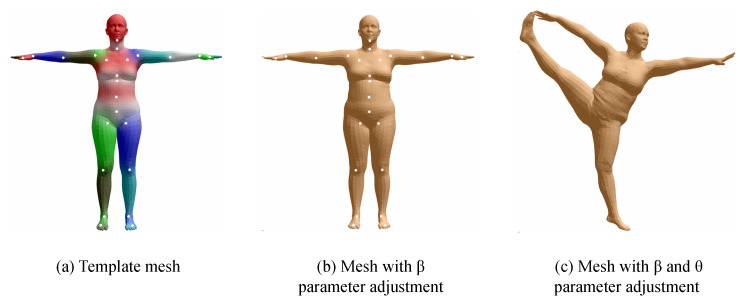
SMPL model parameter adjustment: (**a**) The base SMPL mesh, (**b**) the β parameter modifies body proportions, (**c**) the β parameter defines the body structure, and the θ parameter controls the rotation of 24 joints. Part of the figure is from [23].

The volumetric model represents the human body using parameterized human body models or triangulated meshes, providing an approximate representation of muscle shapes on the skeletal structure. A widely used parametric representation of the human body is SMPL, a parametrically adjustable human model proposed by the Max Planck Institute for Intelligent Systems in Germany [23], which contains 24 joint points and two sets of controllable input parameters, β and θ, where β is the shape adjustment parameter, which consists of a total of 10 parameters responsible for adjusting the height, body size, etc., and the θ group is the pose adjustment parameter, which contains 75 parameters, respectively, responsible for adjusting the relative angles of the 24 joints in 3D space. When using the SMPL model, the first step is to control the shape of the human body through the β parameter, as shown in (a) in Figure 3. The second step is to control the rotation angle of the 24 joints through the θ parameter, as shown in (b) in Figure 3 to adjust the model into the desired pose.

Pavlakos et al. [24] proposed the SMPL-X model as a further improvement of the SMPL model. The SMPL-X model uses more vertices to model the human body in a fine-grained way and incorporates parametric control of facial expression and hand pose. Human body models have made significant advances in recent years, including SoftSMPL [25], STAR [26], BLSM [27], GHUM [28], and others.

### 2.3. Comparison

The kinematic model typically has higher real-time performance and computational efficiency, but may have limitations for complex poses and motion. The volumetric model is usually more detailed and can capture the shape and posture of the human body more accurately, but it has a higher computational complexity. A comparison of the kinematic model and the volumetric model is summarized in Table 2. The selection of a human body model in practice relies on factors like the application scenario, performance demands, and relevant considerations.

## 3. Three-Dimensional HPE from RGB Images

Three-dimensional HPE from RGB images seeks to predict the 3D spatial locations of body joints from RGB images. RGB images provide an appearance of the human body without depth information, thus making it challenging to directly acquire 3D coordinates. Three-dimensional HPE from RGB images is categorized, based on the number of people, into single-person and multi-person settings.

### 3.1. Single-Person 3D HPE

Single-person methods can be further categorized into end-to-end estimation methods and 2D-to-3D lifting methods, which are described below.

#### 3.1.1. End-to-End Estimation

**Figure 4:**
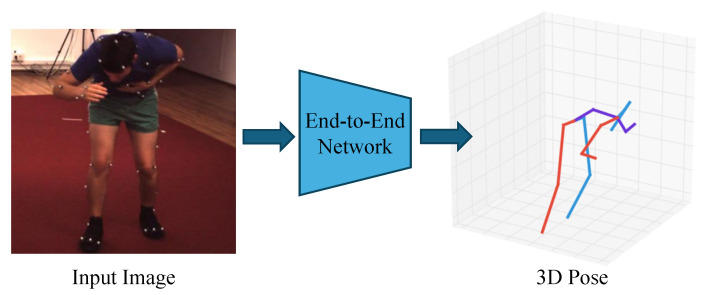
Illustration of end-to-end estimation, where an input RGB image is directly mapped to a 3D pose using deep learning models.

As shown in Figure 4, end-to-end estimation methods directly output the corresponding 3D pose information from the input images. In end-to-end estimation methods, deep learning models such as CNNs are typically employed to learn the mapping from images to 3D poses. These models extract image features and output corresponding pose estimations.

Li et al. [29] pioneered the utilization of neural networks for 3D HPE from a single image. The work first used an eight-layer network to perform the object detection task, then the CNN layer used for feature extraction was repurposed as an initialization model for 3D HPE, with the removal of the head portion of the object detection network during training. Park et al. [30] also used a CNN for 3D HPE, proposing a network architecture similar to [29]. The distinction is that this network used 2D pose information as an auxiliary input instead of using only monocular images, utilizing the 2D pose information to enhance the results of 3D HPE. Mehta et al. [31] employed transfer learning to migrate the knowledge learned from 2D HPE to 3D HPE. Similarly, Zhou et al. [32] proposed a weakly supervised transfer learning approach that utilized both 2D and 3D labels in combination. The 2D HPE subnetwork and the 3D depth regression subnetwork shared the same features, enabling 3D labels from indoor environments to be transferred to outdoor environments. Due to the structure of capsule networks [33], capsule networks have demonstrated the capacity to model the geometric properties of training data. Ramirez et al. [34] solved the ill-posed regression issue of estimating 3D pose from RGB images based on the dynamic vector capsule network presented in [33]. However, the work of Ramirez et al. [34] only utilized lateral viewpoints from the Human3.6M dataset. Garau et al. [35] used matrix capsules [36] to cope with camera view changes. They argued that matrix capsules are more suitable for 3D HPE than vector capsules, with greater generalization ability when changing camera view.

#### 3.1.2. Two-Dimensional-to-Three-Dimensional Lifting

**Figure 5:**
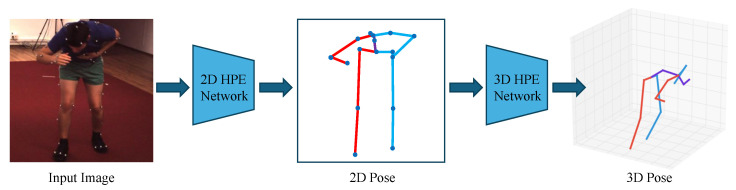
Illustration of 2D-to-3D lifting, where 2D keypoints are first detected and then mapped to 3D space.

Inspired by the recent achievements in 2D HPE, 2D-to-3D lifting methods have gained popularity as a viable solution for 3D HPE. The 2D-to-3D lifting methods first estimate the positions of 2D keypoints from the input image and then map the 2D keypoints to 3D space, as shown in Figure 5. Zhao et al. [37] utilized intermediate visual representations from pre-trained 2D pose detectors, leveraging the joint-centric spatial context encoded within these representations to significantly enhance the accuracy of 3D pose estimation. In the 2D keypoint detection stage, CNN-based keypoint detectors are commonly used, such as OpenPose [38] and hourglass [39]. In the 3D pose reconstruction stage, optimization methods such as adding geometric constraints to the deep learning model can be utilized to map 2D keypoints to 3D space.

1. CNN-Based Methods

CNN-based methods have played a significant role in 2D-to-3D HPE by leveraging hierarchical feature extraction to model spatial dependencies. Chen et al. [40] divided 3D HPE into two parts: 2D HPE and mapping 2D keypoints to 3D space. The 2D pose was first estimated using Convolutional Pose Machines (CPMs), and then the nearest-neighbor matching method was used to find the closest 3D pose in the training set. This method achieves satisfactory accuracy only when the training set is sufficient in size and contains a rich variety of actions. Martinez et al. [41] utilized 2D HPE in the first stage and integrated two fully connected networks (FCNs) to estimate the 3D keypoints in the second stage. However, due to the over-reliance on the results of 2D HPE, the performance of 3D HPE may deteriorate when the accuracy of 2D HPE is not accurate enough. The above attempt [41] only used FCN to map 2D keypoints to 3D space, with suboptimal performance. To mitigate this problem, researchers have started to incorporate geometric dependencies into the network to enhance the accuracy of 3D HPE. By utilizing the geometric constraints inherent in the human body structure, the accuracy of 3D HPE can be enhanced. Nie et al. [42] proposed a sequential bidirectional recursive network to capture the kinematic and geometric relationships within the human body. Kundu et al. [43] proposed an unsupervised 3D pose estimation framework that preserves kinematic structure with minimal prior knowledge of the 3D kinematics. Moreno-Noguer et al. [44] innovatively used the N×N Euclidean distance matrix (EDM) to represent 2D and 3D human poses and transformed the pose estimation problem into a matrix regression problem.

CNN-based approaches generally excel in extracting local spatial features due to their convolutional receptive fields, making them effective in detecting joint locations and preserving fine-grained details in pose estimation. However, their reliance on fixed-sized kernels imposes limitations in capturing global structural relationships, which are crucial in modeling the interdependencies among human joints. While deeper networks with larger receptive fields can somewhat alleviate this limitation, they come at the expense of significantly increased computational cost and memory consumption, making real-time applications less feasible. Furthermore, standard CNNs utilize fixed-size convolutional kernels for local feature extraction, making them effective in capturing short-term dependencies. However, when modeling long-range dependencies, CNNs require stacking multiple convolutional layers to expand the receptive field. This not only leads to increased computational overhead but also makes the network more susceptible to vanishing or exploding gradients, ultimately restricting the effective propagation of information over long temporal sequences. Improvements to CNN-based time-series modeling that address these limitations are discussed in greater detail in Section 4.

2. GCN-Based Methods

**Figure 6:**
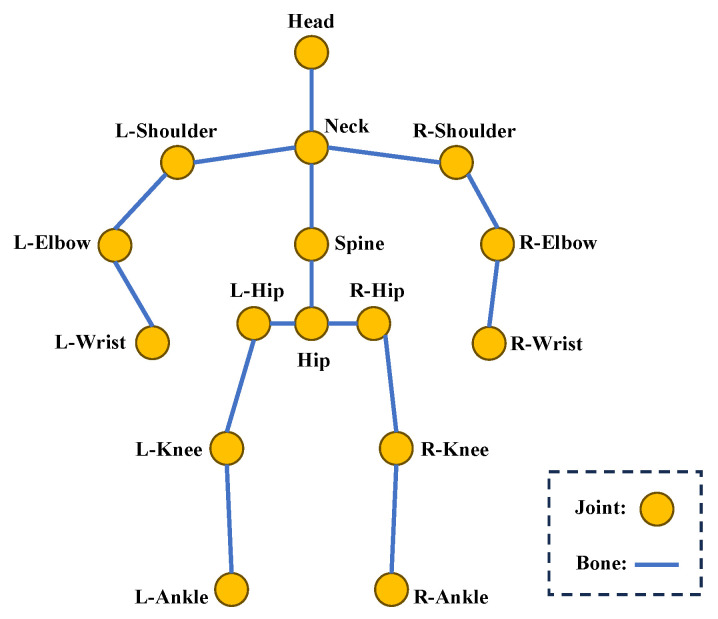
Topology of the human skeleton.

Since the topology of the human skeleton can be depicted as a graph, with joints serving as nodes and the connections between bones as edges, as shown in Figure 6, many studies [45,46,47] have adopted GCNs for lifting 2D poses to 3D. Zhao et al. [45] proposed SemGCN, which incorporates semantic-aware graph convolutions and non-local blocks to capture both local and global joint dependencies for accurate 3D pose estimation. Compared to a CNN, which operates on grid-like data with fixed local receptive fields, a GCN naturally adapts to the irregular structure of human skeletons. By explicitly modeling joint-to-joint relationships through graph edges, a GCN can capture both spatially close and distant dependencies, enabling more effective feature aggregation across the entire skeleton.

One limitation of GCNs is that they share the feature transformations of each node within the graph convolution layer. Although weight sharing can reduce parameters and enhance computational efficiency, for human modeling, it limits the model’s flexibility to capture diverse human structures and motions. Liu et al. [46] conducted an inaugural comprehensive analysis of weight sharing in GCNs and explored the impact of weight sharing in different layers and different network structures on the performance of 3D pose estimation. They used pre-aggregation to achieve no weight sharing, implementing distinct transformations on the input features of each node before they were aggregated. Nonetheless, it notably enlarged the size of the model. Zou et al. [47] proposed a modulated GCN, which differs from pre-aggregation [46] in that weight modulation uses the same shared weight matrix but learns different modulation vectors for each node, enabling the model to distinguish between different joint connections while keeping its size compact. Ci et al. [48] extended GCNs by removing weight sharing and decoupling the structural and transformation matrices, resulting in a locally connected graph network that better captures joint-specific features. Chen et al. [49] modeled the channel topology by learning the shared topology as a common prior for all channels and refining it by channel-specific correlations for each channel.

**Figure 7:**
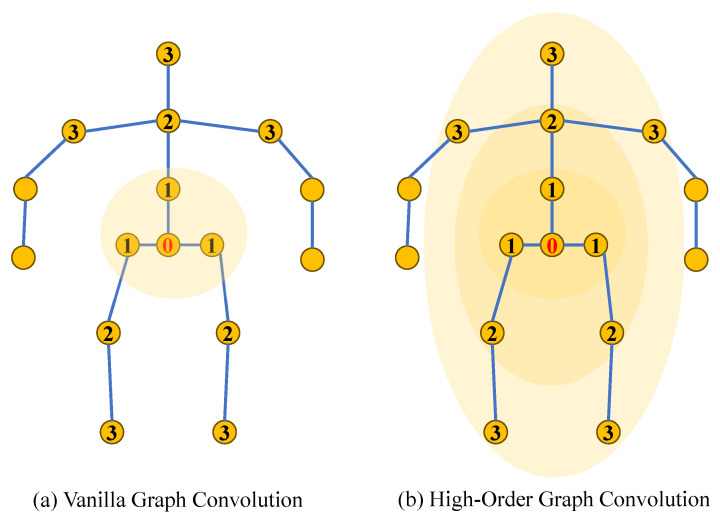
Contrast between a vanilla graph convolution and a high-order graph convolution focused on a skeletal graph structure. The numbers indicate the distance from the root node (0): 1 for first-order, 2 for second-order, and 3 for third-order neighbors.

Furthermore, GCNs mainly focus on understanding connections between body joints through first-order neighbors, as shown in Figure 7, overlooking higher-order neighbors, which limits their ability to discern relationships between joint nodes that are further apart. Recent models have focused on building intricate higher-order relationships among joints to represent skeleton information more effectively. Zou et al. [50] aggregated features from nodes at different distances to capture remote relationships between body joints. Quan et al. [51] used multi-hop neighborhoods for node feature aggregation to capture remote dependencies between joints. Li et al. [52] proposed an architecture that integrates multi-layer perceptrons (MLPs) [53] with a GCN through SG-MLP and CG-MLP blocks, effectively capturing both global dependencies and local structural relationships in human poses.

3. Diffusion-Based Methods

Generative models, including generative adversarial networks (GANs) [54,55], variational autoencoders (VAEs) [56,57,58], and diffusion models [59], have been extensively investigated for structured prediction tasks such as 3D HPE. Since monocular 3D HPE involves reconstructing 3D poses from inherently ambiguous 2D observations, generative models play a crucial role in capturing the underlying distribution of plausible 3D poses. Unlike direct regression-based approaches such as CNNs and GCNs, which predict a single deterministic pose, generative models are capable of producing diverse and realistic pose hypotheses, making them particularly valuable for addressing depth ambiguity and occlusion in monocular settings. GANs leverage an adversarial framework, where a generator learns to produce realistic samples while a discriminator distinguishes between real and synthesized data. While GANs have shown promise in generating high-fidelity samples, they often suffer from mode collapse and instability, limiting their ability to capture the full diversity of human poses. VAEs, on the other hand, rely on probabilistic latent space representations to generate structured outputs, but their assumption of Gaussian priors frequently leads to oversmoothed reconstructions that lack fine-grained details, making them less suitable for precise 3D pose estimation.

**Figure 8:**
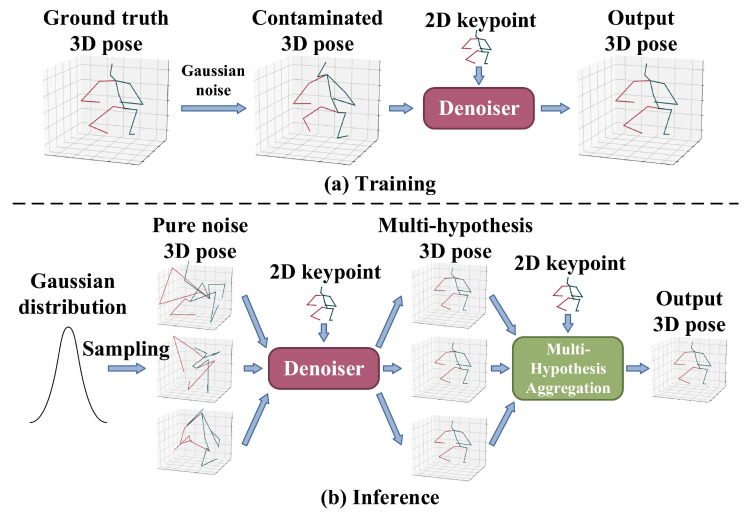
Formulating 3D HPE as a reverse diffusion process. (**a**) Training: the ground-truth 3D pose was destroyed and reversed using a denoiser for a clean 3D prediction. (**b**) Inference: Gaussian noise is used with 2D keypoints to generate 3D pose hypotheses through the denoiser. Part of the figure is from [61].

In diffusion-based 3D HPE, ground-truth 3D joints are progressively corrupted with Gaussian noise during the forward diffusion process. The model is trained to reverse this process through iterative denoising conditioned on 2D keypoints, allowing for the structured generation of diverse and anatomically plausible 3D pose hypotheses. Unlike GANs, diffusion models do not require adversarial training, thus avoiding mode collapse and training instability, while also surpassing VAEs by generating sharper and more realistic samples without being constrained by restrictive latent space assumptions. Their iterative refinement mechanism enables them to generate multiple plausible hypotheses for 3D human poses, which is particularly advantageous in monocular settings where occlusions and depth ambiguity pose significant challenges. Gong et al. [60] and Shan et al. [61] formulated 3D HPE as a reverse diffusion process in a diffusion model, as shown in Figure 8; Gaussian noise is added to the ground-truth 3D joint coordinates during the forward diffusion process, progressively transforming clean poses into noise through a variance schedule. In the reverse process, the model learns to reconstruct the original pose from the noisy sample conditioned on the corresponding 2D keypoints. The entire framework is supervised by a mean squared error (MSE) loss between the reconstructed and ground-truth 3D poses. During inference, multiple 3D pose hypotheses are generated by sampling initial poses from a Gaussian distribution and applying the reverse diffusion process through the denoiser. Jiang et al. [62] leveraged the pre-trained diffusion-based model for generating 3D human poses to tackle the challenges associated with cross-domain and in-the-wild 3D HPE, where they iteratively optimized target 3D poses through an optimizer that calculated optimal translations, integrated with the denoising steps of the diffusion process. Cai et al. [63] proposed a disentangled diffusion-based method called DDHPose, which decomposes the 3D joint location into bone length and bone direction, adding noise during the forward process. In the reverse process, the noisy bone length, noisy bone direction, and 2D pose are fed into the HSTDenoiser, which contains HRST and HRTT modules to reverse the 3D pose from the noisy input by denoising in both spatial and temporal dimensions. Ji et al. [64] employed a diffusion model to establish a credible distribution for joint locations in both 3D and 2D spaces, resulting in an innovative method for pose enhancement. Xu et al. [65] presented a new fine-grained prompt-driven denoiser based on a diffusion model for 3D HPE named FinePOSE, which integrates diffusion models with a prompt-driven mechanism. Leveraging the generative capabilities of diffusion models, FinePOSE progressively refines noise into high-quality 3D poses. The fine-grained prompts further optimize the generation process, enabling the model to excel in handling complex and intricate poses with exceptional precision.

Despite their remarkable performance, diffusion models still present challenges in computational efficiency due to their iterative denoising process, which requires multiple forward passes to generate final predictions. Recent efforts to accelerate inference have explored techniques such as Denoising Diffusion Implicit Models (DDIMs) [66] and score distillation sampling (SDS) [67] to reduce the number of sampling steps while maintaining high-quality reconstructions. Future research should focus on optimizing inference speed, leveraging self-supervised learning for improved generalization.

#### 3.1.3. Comparison

End-to-end estimation and 2D-to-3D lifting have their own advantages and limitations, summarized in Table 3. End-to-end estimation benefits from the rich information contained in images and enables end-to-end training and inference. Nonetheless, it can be influenced by numerous factors, including background, lighting, etc. A network trained on a specific dataset often struggles to adapt to other datasets across diverse settings, like indoor versus outdoor scenarios. The advantage of 2D-to-3D lifting is the use of geometric constraints and projection models, which can better utilize the geometric relationship between 2D keypoints and 3D space. This approach can be more flexible in dealing with 2D keypoints detection and 3D pose reconstruction, and different algorithms and models can be selected according to the requirements. However, 2D-to-3D lifting is highly dependent on the precision of detecting 2D keypoints. Mistakes in estimating the 2D pose can cascade into the 3D model, resulting in erroneous 3D poses.

In the 2D-to-3D lifting framework, the design of the 3D estimation module plays a critical role in determining the overall performance. Common architectural choices include CNNs, GCNs, and diffusion models, which serve as the backbone for lifting 2D keypoints into 3D space. CNN-based methods excel at capturing local spatial features and joint locations but struggle with modeling global structural relationships due to their limited receptive fields. GCN-based methods naturally align with the graph-like topology of the human skeleton and can effectively model joint dependencies. However, they may be limited by weight sharing and reliance on first-order neighbors, which constrain their ability to capture joint-specific and higher-order structural relationships. Diffusion-based methods model 3D pose estimation as a reverse denoising process, adding Gaussian noise to ground-truth poses during training and learning to recover clean poses from noisy inputs conditioned on 2D keypoints, enabling the generation of diverse 3D hypotheses. While offering superior accuracy, diffusion-based methods are more computationally intensive than CNNs and GCNs due to their iterative denoising process, which requires multiple forward passes to gradually refine noisy inputs into clean poses, making them less suitable for real-time applications without dedicated acceleration techniques.

### 3.2. Multi-Person 3D HPE

Given that the number of people within an image is unknown, the first problem that the algorithm needs to solve is how to estimate multiple human body poses with unknown positions and unequal scales at the same time, which is referred to as the multi-person pose estimation problem. To cope with this problem, researchers have proposed a variety of approaches, but most of them follow one of the following two paradigms: top-down and bottom-up.

#### 3.2.1. Top-Down Methods

**Figure 9:**
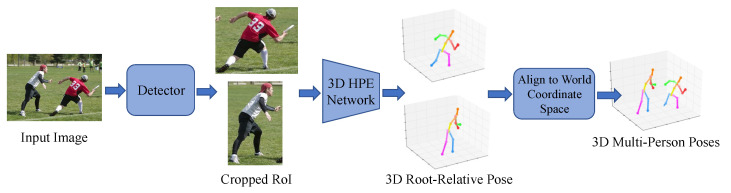
Illustration of top-down methods: Human regions are first detected and cropped, followed by the estimation of 3D root-relative poses, which are then aligned to the world coordinate space. Part of the figure is from [68].

Top-down approaches initially identify individual human areas using a human detection network, then apply a 3D pose network to each detected area to estimate the 3D pose, as shown in Figure 9. Moon et al. [68] proposed a method where cropped human images are analyzed by a root localization network to estimate human root positions relative to the camera. A single-person pose network then estimates the 3D pose for each person.

Top-down methods tend to have more serious errors in some examples of poor segmentation due to their dependence on the results of the bounding box for object detection. For example, errors in detecting a single person’s bounding box can lead to incomplete keypoint capture, so most top-down methods have evolved to solve this problem. Benzine et al. [69] proposed a pose-aware anchor selection strategy to solve the multi-person overlap problem by discarding unambiguous anchors during inference. Top-down methods typically make a single assumption about the person in the bounding box. However, such a single assumption can limit their performance in crowded scenes with occlusion. To address this challenge, Khirodkar et al. [70] predicted several 2D pose instances inside a single bounding box. In addition, the global information in the image may be ignored as top-down methods initially detect the bounding box of each person. Wang et al. [71] addressed the lack of global information by encoding interaction details with hierarchical depth and angle ordinal relations, capturing semantic details at body-part and joint levels for global coherence. GCNs excel in multi-person 3D pose estimation by modeling interactions between multiple skeletons within a unified graph. Qiu et al. [72] proposed GR-M3D, a dynamic graph reasoning approach that adaptively adjusts decoding graphs based on input images. By extracting multiple root keypoints and dynamically weighting decoding paths, GR-M3D enhances robustness to occlusions and depth ambiguities, improving multi-person pose estimation accuracy.

#### 3.2.2. Bottom-Up Methods

**Figure 10:**
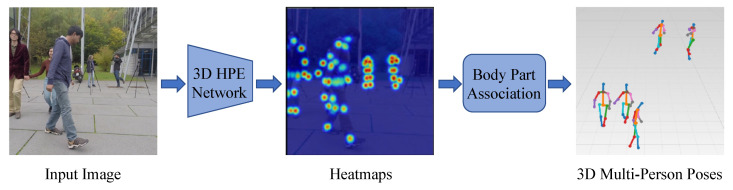
Illustration of bottom-up methods, which first detect all joints in an image and then cluster them to form individual human skeletons. Part of the figure is from [73].

Bottom-up methods consist of two main parts: joints detection and joint clustering. In joint detection, all keypoints of all people in the picture need to be detected, and then the detected joints are clustered, corresponding to the generation of the human skeleton of each single person, as shown in Figure 10.

The two main challenges faced in bottom-up methods are joint clustering and occlusion. The problem with joint clustering is that in scenarios with multiple people, there may be mutual interference or confusion between joints. For example, when multiple human bodies have joints in close proximity, it may be difficult to accurately group the corresponding joints into the correct body. Zanfir et al. [74] proposed MubyNet, which integrates 2D keypoint detection, person grouping, and 3D pose estimation within a unified framework. The method formulates body part grouping as a constrained binary integer programming problem to enforce kinematic consistency and employs attention-guided modules to enhance 3D pose estimation accuracy. Fabbri et al. [75] introduced compressed volumetric heatmaps by combining a heatmap autoencoder with a code predictor, enabling efficient and scalable 3D pose inference with consistent runtime regardless of the number of people. The issue of occlusion arises frequently in multi-person scenarios, especially when there are intersections or significant occlusions between human bodies. Zhen et al. [76] proposed a depth-aware part association algorithm for inferring person-to-person occlusion and bone length constraints from predicted body depths. Liu et al. [77] utilized two distinct encoders to divide the reasoning task into two components: reconstruction and imitation. The first encoder pinpointed crucial information for reconstructing occluded joints, while the second focused on extracting visible cues. Mehta et al. [78] proposed a fully convolutional network that directly regresses 2D keypoints and root-relative 3D joint positions using occlusion-robust pose maps. By redundantly encoding complete 3D poses across multiple visible joints, this approach improves robustness to occlusion and truncation.

#### 3.2.3. Comparison

Top-down methods rely on the results of object detection and single-person pose estimation methods, which are more robust to different scales of the human body and usually have higher accuracy, but the computational complexity increases with the number of people. In addition, the global information in the image may be ignored as top-down methods initially detect the bounding box of each person. Conversely, bottom-up methods have linear computational complexity. Nonetheless, they may face challenges in grouping joints in highly overlapping or occluded multi-person scenarios, and errors may occur when estimating people on a small scale. A comparison of top-down and bottom-up approaches is summarized in Table 4. To address these challenges comprehensively, Cheng et al. [73] integrated top-down and bottom-up methods to leverage their respective strengths. The top-down network collectively estimates joints from all individuals to enhance robustness against potential erroneous bounding boxes. Meanwhile, the bottom-up network incorporates human detection-based normalized heatmaps, making it robust to scale variations. The 3D poses estimated by both methods are then integrated for the final pose results.

## 4. Three-Dimensional HPE from Videos

**Figure 11:**
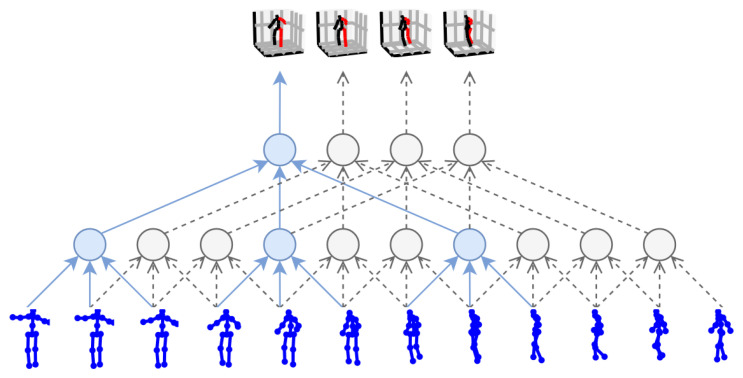
Three-dimensional HPE from videos takes 2D keypoint sequences as input and generates 3D pose estimates as output. Part of the figure is from [80].

While considerable advancements have been achieved in 3D HPE from images, translating these advancements directly to video sequences often fails to yield the expected results. To alleviate such problems and improve accuracy and robustness, many methods [9,79,80] have integrated temporal information from 2D pose sequences to explore the spatial and temporal correlations of joints, as shown in Figure 11.

### 4.1. Recurrent-Based Methods

Recurrent neural networks (RNNs) are a widely used recurrent-based architecture for processing time-series data, designed to retain memory and capture long-term dependencies within a sequence. In 3D HPE from videos, RNNs can aid in understanding the pose variations between frames and model the continuity and coherence of motions within the temporal data. Lin et al. [81] combined a CNN with an RNN in a multi-stage process to learn image structures and sequence contexts. However, standard RNNs often suffer from problems such as gradient vanishing or gradient explosion when backpropagated through time steps. To overcome these limitations, long short-term memory (LSTM) uses gating mechanisms and a cell state to regulate information flow. Lee et al. [82] improved the estimation of joint positions by propagating information through LSTM units at each time step based on the current joint estimation results and the dependencies between joints. Hossain et al. [83] designed a network with layer-normalized LSTM units and imposed a temporal smoothness constraint during training.

### 4.2. TCN-Based Methods

In video-based human pose estimation tasks, TCNs offer significant advantages over RNNs and LSTMs. As an enhanced version of CNNs for time-series modeling, TCNs mitigate some of CNNs’ limitations in capturing long-range dependencies through dilated convolutions and residual connections. Dilated convolutions introduce gaps between convolutional kernel elements, allowing the network to expand its receptive field without increasing computational overhead, thereby enabling more effective integration of long-term information. Pavllo et al. [80] proposed the classic TCN-based 3D HPE method called VideoPose3D, which takes a sequence of 2D poses as input. The model employs a residual TCN architecture combined with dilated convolutions to expand the receptive field, enabling the processing of temporal information and capturing long-term dependencies. Liu et al. [84] extended TCNs by incorporating an attention mechanism, allowing the model to adaptively identify key frames within the sequence. Shan et al. [85] employed a TCN as the backbone local feature encoder for extracting features from grouped 2D keypoints but noted its limitation in treating all frames equally. To overcome this, they proposed a temporal information encoding method to enhance the effectiveness of TCNs in modeling inter-frame relationships and capturing fine-grained motion dynamics. Overall, TCNs surpass RNNs and LSTMs in efficiently modeling long-range dependencies and enabling parallel computation while also partially overcoming CNNs’ limitation of a restricted local receptive field. However, they still inherits CNNs’ drawback of lacking explicit global temporal modeling. To better capture long-range dependencies, integrating attention mechanisms or graph neural networks could further enhance their capability in global sequence modeling. Yuan et al. [86] proposed GTA-Net, which combines Joint-GCN and Bone-GCN for spatial relation modeling, and employs a hierarchical attention-augmented TCN to effectively capture temporal dependencies and motion dynamics.

### 4.3. Transformer-Based Methods

In recent years, Vision Transformers (ViTs) [87] have made significant progress in visual sequence modeling. The transformer architecture [88] introduces a self-attention mechanism, allowing it to simultaneously process every position in the input sequence without being limited by the length of the sequence. This characteristic helps to solve the gradient vanishing and gradient explosion problems, thus enhancing the stability of training. Zheng et al. [9] proposed the first fully transformer-based 3D HPE method that does not involve a convolutional architecture. The spatial transformer module focuses on understanding the connections between joints, and the temporal transformer module is designed to capture the overarching connections across the entire sequence of frames. Building on this spatial–temporal modeling paradigm, subsequent works have aimed to further improve the effectiveness and efficiency of joint space–time representation. For example, Zhang et al. [89] proposed MixSTE to use temporal transformer and spatial transformer alternatively to obtain better spatial–temporal feature encoding. Li et al. [90] proposed using a spatial transformer to generate multiple hypothesis representations for poses, followed by different temporal transformer blocks to model multi-level global correlations. Wang et al. [91] and Zhao et al. [92] proposed CrossFormer and GraFormer, respectively, both of which modeled dependencies between body joints but focused mainly on pairwise interactions of body joints, which falls short in scenarios with overlapping joints and fast pose changes. To overcome these limitations, subsequent methods have introduced hierarchical modeling strategies to capture more structured and robust representations. Chen et al. [93] proposed HDFormer, which combines self-attention and higher-order attention to formulate a multi-order attention module that utilizes higher-order bone and joint relationships to improve pose estimation. Mehraban et al. [94] introduced AGFormer, which employs two parallel streams, one utilizing a transformer to capture global joint interdependencies, and the other utilizing GCNFormer to capture local joint relationships, enabling effective integration of both global and local information. Wei et al. [95] proposed a lightweight multi-scale transformer architecture called PGFormer, which incorporates a Pyramid Graph Attention (PGA) module to model hierarchical long-range dependencies by capturing cross-scale correlations among joints and body parts. Peng et al. [96] proposed KTPFormer, incorporating Kinematics Prior Knowledge Enhancement (KPA) and Trajectory Prior Knowledge Enhancement (TPA) modules. KPA models body kinematics, while TPA captures joint motion trajectories, enabling efficient spatial–temporal modeling. These lightweight modules can be seamlessly added to transformer-based networks with minimal computational cost.

One of the key features of the transformer is the self-attention mechanism, which enables the model to capture long-range dependencies but also leads to high computational costs. At each position, each attention head requires computation proportional to the length of the sequence, and thus the computational cost increases significantly at larger sequence and model sizes. Einfalt et al. [97] proposed Uplift and Upsample, which reduces computational complexity by using temporally sparse 2D inputs and masked token modeling for upsampling, achieving a twelvefold speedup in inference time while remaining competitive with SOTA methods. Li et al. [79] proposed Stride Transformer to boost long 2D pose sequences into single 3D poses, which reduces sequence redundancy and computational cost. Zhao et al. [98] proposed PoseFormerV2, which encodes skeleton sequences into low-frequency DCT coefficients, preserving motion information while reducing token count and efficiently expanding the receptive field. Li et al. [99] proposed a plug-and-play pruning-and-recovering framework called HoT. HoT includes a TPC module to select a few representative tokens for video redundancy reduction and a TRA module to restore the original temporal resolution for fast inference. Experiments on Human3.6M show that HoT reduces FLOPs by nearly 50% without accuracy loss, and by 40% with only a 0.2% accuracy drop.

### 4.4. Mamba-Based Methods

Mamba [100] is a recently proposed sequence modeling method based on the SSM, designed for efficient long-range dependency modeling with low computational cost. Compared to traditional RNNs and LSTMs that suffer from sequential bottlenecks, and transformers with quadratic self-attention complexity, Mamba leverages parameterized state-space equations to enable efficient parallel training, linear-time inference, and reduced memory consumption. While Mamba-based methods demonstrate notable advantages in efficiency, they may exhibit limitations in scenarios that demand fine-grained spatial reasoning, where self-attention mechanisms or graph-based methods tend to perform better. In addition, due to the simplified nature of state-space representations, modeling complex non-local interactions in highly articulated human poses may be less expressive compared to fully attention-based models. Nevertheless, recent studies [101,102] have demonstrated that these limitations can be effectively mitigated through well-engineered architectures, such as the integration of Mamba with GCNs and the incorporation of global–local information fusion strategies. As a result, Mamba-based models are capable of achieving performance comparable to or even exceeding that of more computationally demanding baselines, while simultaneously preserving their advantages in inference efficiency, thereby making them particularly suitable for real-time and resource-constrained applications. Huang et al. [101] pioneered the application of Mamba to 3D HPE by proposing a novel framework named PoseMamba, which is based on a bidirectional global–local spatio-temporal SSM. The framework incorporates a bidirectional global–local spatio-temporal Mamba block to comprehensively model the relationships between human joints within each frame as well as the temporal dependencies across frames. Li et al. [103] proposed SMGNFormer, which utilized the Mamba architecture to extract spatial information from 2D keypoints and combined GCNs with multi-head attention to build a relational graph of 2D keypoints. The outputs are subsequently processed by a time–frequency feature fusion transformer to estimate 3D human poses. Zhang et al. [102] proposed Pose Magic, a hybrid Mamba-GCN architecture for 3D human pose estimation, which enhances local dependencies using GCN while leveraging Mamba for efficient long-range modeling. By adaptively fusing both representations, Pose Magic achieves new SOTA results with 74.1% fewer FLOPs and improved motion consistency.

### 4.5. Comparison

RNNs are one of the earliest neural network architectures developed for sequence modeling. Their main strength is processing variable-length sequences and passing information through hidden states. Due to their simple structure, RNNs are well suited for tasks involving short sequences, such as text classification and basic time-series prediction. However, RNNs may also suffer from problems such as gradient vanishing or gradient explosion when backpropagated through time steps.

LSTM is an improved version of an RNN, which addresses the vanishing gradient problem by introducing gating mechanisms and a cell state. LSTM is better at capturing long-range dependencies, making it suitable for tasks like machine translation and speech recognition. However, like an RNN, it cannot process sequences in parallel, resulting in slower training.

A TCN is a convolution-based sequence modeling approach that leverages dilated convolutions and residual connections to effectively capture long-range dependencies while maintaining computational efficiency. Unlike RNNs and LSTM, TCNs enable fully parallel processing, leading to faster training and inference. By stacking dilated convolutions, TCNs can expand their receptive field exponentially without requiring deeper networks, mitigating the gradient vanishing problem associated with recurrent architectures. However, TCNs treat all time steps equally and lack explicit mechanisms to model dynamic relationships between frames, which may limit their adaptability in complex sequence modeling tasks.

Transformer completely abandons the step-by-step computation of RNNs and LSTM by using the self-attention mechanism, enabling parallel processing of entire sequences and significantly improving training efficiency. Transformer excels at capturing long-range dependencies and is suitable for long-sequence tasks. However, the computational complexity of transformers is $O(N^{2})$ (where *N* is the sequence length), leading to high computational costs for extremely long sequences.

Mamba is a sequence modeling method based on the SSM, combining the sequence modeling strengths of RNNs with the efficient parallelization of transformer. It offers high computational efficiency with a complexity of O(N), making it suitable for ultra-long sequences, while its state transition equations enable effective long-range dependency modeling without gradient vanishing issues. Additionally, the memory efficiency of Mamba with linear memory usage growth and no need for attention matrices makes it ideal for resource-constrained scenarios.

A comparative analysis of architectural characteristics for video-based 3D human pose estimation is summarized in Table 5, while quantitative performance benchmarking of these architectures is presented in Table 6.

## 5. Datasets and Evaluation Metrics

Datasets play a crucial role in facilitating HPE research by providing standardized benchmarks for algorithm evaluation and comparison. This section outlines the most commonly used datasets and evaluation metrics for 3D HPE.

### 5.1. Datasets

**Figure 12:**
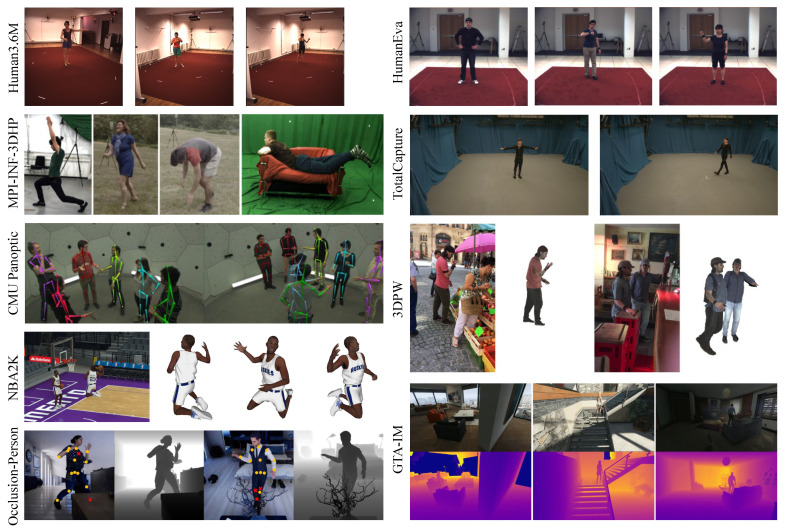
Examples from commonly used datasets.

In 3D HPE, datasets are mainly categorized into real and synthetic datasets. Real datasets are measured or collected directly from real-world environments, and synthetic datasets are synthesized artificially using computers. Real datasets offer a more accurate reflection of human poses in real scenes, including real human shapes, motions, and environments. The disadvantages of using real datasets in 3D HPE are outlined as follows: (1) Obtaining human pose information requires motion capture systems such as MoCap and IMUs. As a result, many 3D human pose datasets are produced in restricted environments, leading to limited diversity in dataset scenes. (2) Real datasets may contain personally identifiable information that requires privacy-preserving processing, which may lead to limited openness of the datasets. (3) The frequency of occurrence of different types of motions and scenarios may be unbalanced, leading to poor performance of the model in certain scenarios. Synthetic datasets are often produced using game engines in 3D HPE, where synthetic data can effectively reduce cost and time while avoiding privacy and ethical issues. The disadvantage of synthetic datasets is that, when applied to the real world, they may suffer from domain gap issues that can affect performance. A combination of these two types of datasets is used in many studies to leverage their advantages and mitigate their respective limitations. Table 7 lists the commonly used datasets in 3D HPE, and Figure 12 illustrates examples of the datasets.

1. HumanEva

The HumanEva [22] dataset was produced using motion capture equipment and proposed by Sigal L et al. in 2010. The dataset records six daily actions of four actors, such as walking, jogging, boxing, greeting, etc. The dataset contains a total of 20,610 labeled images, typically using actions from individuals S1, S2, and S3 for training, with the remaining samples for testing. HumanEva is one of the earlier proposed datasets for 3D HPE and motion analysis, and although it may be relatively limited in scale and diversity, it served as a starting point for researchers when it was introduced. Researchers need to be aware of its limitations when using this dataset. For broader and more in-depth research, consideration should be given to comparing and integrating it with other larger-scale datasets.

2. Human3.6M

The Human3.6M [21] dataset, abbreviated as H36M dataset, was proposed by Ionescu et al. in 2014. The H36M dataset comprises approximately 3.6 million images recorded with motion capture devices on a total of 11 actors, including 6 males and 5 females. The recording of the actors is conducted from four different viewpoints. Each actor performs 17 daily activities, such as smoking, taking photos, greeting, and more. Researchers usually use the action samples of S1, S5, S6, S7, and S8 as the training set and the action samples of S9 and S11 as the test set. The H36M dataset is currently the largest and most widely used dataset in the field of 3D HPE.

3. CMU Panoptic

The CMU Panoptic [106] dataset was proposed by Joo et al. in 2016 for markerless motion capture using a multi-view system. It contains 65 sequences (5.5 h) of social interactions with 1.5 million 3D skeletons. The CMU Panoptic dataset provides rich viewpoint information, and the sequences in the dataset capture the interactions of multiple people in social scenarios, which is important for studying multi-person pose analysis and collaborative actions. The multi-view information contained in the dataset also adds to the complexity of data processing and computational costs.

4. MPI-INF-3DHP

The MPI-INF-3DHP [31] dataset, abbreviated as MPII dataset, is a 3D HPE dataset consisting of indoor and outdoor scenes, proposed by Dushyant Mehta et al. in 2017. The dataset consists of more than 1.3 million frames of images from 14 camera angles, recording eight types of activities from eight participants. The MPII dataset has greater diversity in terms of poses, human appearances, clothing, occlusions, and viewpoints, thus complementing the existing corpus and expanding the scope of enhancement.

5. TotalCapture

The TotalCapture [107] dataset was captured by Trumble et al. in 2017 using eight calibrated HD cameras filming at a frame rate of 60 Hz in an indoor room with a space of approximately 8 m × 4 m. It includes four male actors and one female actor, each performing four distinct actions repeated three times, with a range of movements including ROM, walking, acting, and freestyle. The TotalCapture dataset includes synchronized videos, IMUs, and Vicon data totaling 1,892,176 frames. It encompasses a variety of actions, providing a challenging benchmark for the comprehensive evaluation of algorithm performance.

6. 3DPW

The 3DPW [108] dataset is the first dataset in the wild with precise 3D pose annotations for evaluation, created by von Marcard et al. in 2018. 3DPW is the first dataset that features video sequences captured with a mobile phone camera in motion. The dataset contains more than 51,000 frames with 18 3D models wearing different outfits, containing everyday actions including walking around the city, drinking coffee, or riding the bus. The 3DPW dataset holds significant importance in providing human pose data from real-world scenarios. Despite its relatively small size, its realism and diversity make it valuable for evaluating algorithm performance in real-world scenarios.

7. NBA2K

The NBA2K [109] dataset, created by Zhu et al. in 2020, contains human body mesh and texture data for numerous NBA athletes, each with about 1000 different movements. For each human mesh, 3D poses containing 35 keypoints, such as face and finger, and their corresponding color images and camera parameters are also provided. The dataset contains 27 real ballplayers, but the authors lack permission to disclose these data containing NBA athletes, so a synthetic dataset containing 28 more virtual athletes was constructed, and the entire framework was retrained, with the synthetic athletes having the same geometric and visual quality. The dataset is limited to motions in basketball and may be less relevant for pose estimation in other domains.

8. GTA-IM

The GTA-IM [110] dataset was created by Cao et al. in 2020 as an indoor activity dataset derived from the Grand Theft Auto (GTA) video game virtual engine. It contains 1 million RGB-D frames at 1920×1080 resolution, features 98 3D human joint points with annotations, and covers a variety of actions, including walking, and opening doors. Each scene contains various environments, like living rooms and kitchens, highlighting interactions between humans and their surroundings.

9. Occlusion-Person

The Occlusion-Person [111] dataset was created by Zhang et al. in 2020 using UnrealCV to render multi-view images and depth maps from 3D models. Thirteen models of the human body with different outfits were placed in nine different scenes, such as the living room, bedroom, and office. Objects such as sofas and tables were intentionally used to obscure some body joints. The Occlusion-Person dataset provides images that simulate the occlusion situation in real-life scenarios, which helps researchers explore the performance of the algorithms in complex environments. When Occlusion-Person is used as a virtual dataset along with NBA2K and GTA-IM, researchers need to consider the disparity between the virtual data and the real data when using them. To more thoroughly evaluate the performance of the algorithm, researchers can contemplate combining virtual datasets with real-world datasets to mitigate the limitations of virtual data.

### 5.2. Evaluation Metrics

In 3D human pose estimation, several metrics are used to quantify the accuracy of predicted joint positions. We consider a set of *N* joints. For each joint, $J_{i}$ denotes the predicted position, while $J_{i}^{*}$ represents its corresponding ground-truth position, where *i* ranges from 1 to *N*. The Euclidean distance between any two joint positions is computed using the $L_{2}$ norm, denoted by ${∥\cdot ∥}_{2}$. We briefly introduce the common metrics used in the field below.

1. Mean Per Joint Position Error (MPJPE)

The MPJPE measures the average of the Euclidean distance between all ground-truth joints and the predicted joints in a single image. A lower MPJPE value indicates better performance. The metric is defined as follows:

(1) $$MPJPE=\frac{1}{N}\sum_{i=1}^{N}{|\left|J_{i}-J_{i}^{*}\right||}_{2}$$

Protocol 1, Protocol 2, and Protocol 3 are different joint alignment protocols used in the calculation of MPJPE [80]. Protocol 1 uses MPJPE as an evaluation metric to compute the average Euclidean distance between the predicted and true joint positions. Protocol 2 uses P-MPJPE as the evaluation metric, which incorporates Procrustes analysis on top of MPJPE. Procrustes minimizes the distance between two sets of points through translation, rotation, and scaling. In P-MPJPE, Procrustes alignment is performed first, and then the aligned MPJPE is computed. Protocol 3 uses N-MPJPE as an evaluation metric. N-MPJPE is a version of MPJPE that normalizes the MPJPE by dividing the error by the bone length of the reference skeleton to eliminate the effect between different body sizes and thus better compare performance between different body sizes. The choice of these protocols depends on the specific evaluation requirements. By considering different sources of error, different protocols can offer more comprehensive evaluation results. Therefore, when comparing the MPJPE of different methods, it is common to specify the protocol used.

2. Percentage of Correct Keypoints (PCK)

The PCK measures the percentage of correctly predicted keypoints within a given distance threshold. For each keypoint, the Euclidean distance between its predicted position and the ground-truth position is computed. If this distance is smaller than the threshold, the prediction is considered correct. The equation for calculating the PCK is expressed as follows:

(2) $$PCK=\frac{1}{N}\sum_{i=1}^{N}1\left[{∥J_{i}-J_{i}^{*}∥}_{2}<D\right]\times 100\%$$

Here, *D* is a predefined distance threshold to determine the accuracy of the keypoint prediction. The indicator function $1[{∥J_{i}-J_{i}^{*}∥}_{2}<D]$ returns 1 if the calculated distance is less than *D* and 0 otherwise. The PCK metric is usually calculated based on the body part of the keypoints, e.g., head, shoulders, elbows, wrists, etc. For each body part, the count of correctly predicted keypoints is divided by the overall number of parts to obtain the PCK value for that body part. Then, the PCK values for all body parts are averaged to obtain the final PCK metric. The choice of distance thresholds usually depends on the specific task and characteristics of the dataset. Common thresholds include scale proportions of body parts (e.g., percentage of body part length) or fixed pixel distances. The PCK metric provides a simple and intuitive evaluation method to quantify the accuracy of 3D HPE. However, it has some limitations, such as the inability to capture subtle deviations in the location of keypoints and differences in the importance of different body parts. Therefore, when evaluating the performance of 3D HPE, it is usually combined with other metrics and evaluation methods for a comprehensive evaluation.

While traditional metrics like MPJPE and PCK are widely adopted in benchmark evaluations, real-world deployment of 3D HPE requires addressing challenges beyond controlled datasets. Practical applications often involve dynamic lighting variations, occlusions, cluttered backgrounds, and multi-person interactions, which are not fully captured by existing evaluation protocols. Zhang et al. [112] introduced PoseBench, a benchmark for evaluating pose estimation robustness against real-world corruptions such as blur, noise, compression, lighting variations, and masks. Testing 60 models across human and animal datasets, they found significant performance drops and highlighted key factors like input resolution, pre-training, and data augmentation to improve robustness. Future evaluation metrics should incorporate dynamic lighting variations, occlusions, cluttered backgrounds, and other real-world challenges to bridge the gap between benchmark performance and practical deployment, ensuring 3D HPE models maintain reliability across diverse environments.

Table 8 lists the performance of 3D HPE methods in recent years on the Human3.6M dataset (Protocol 1), using MPJPE as the evaluation metric. Due to the complexity of evaluating 3D HPE, considering factors such as the length of input video sequences and varying levels of difficulty in different challenges, the results in the table represent only a fraction of the overall performance. The performance of methods should be judged based on a comprehensive analysis of the results.

## 6. Challenges

In 3D HPE, scholars are committed to researching 3D HPE from monocular RGB images and videos, because monocular RGB cameras are widely used and cost-effective, being equipped in everyday devices such as smartphones and computers. Hence, this technology presents vast application prospects. Simultaneously, it presents greater challenges. The following focuses on several pain point problems, and a comparison of each method is shown in Table 9.

1. Dataset

The development of artificial intelligence relies heavily on the synergy between datasets and algorithms. Data serve as the foundation for algorithms, and high-quality datasets can significantly improve model performance. There are four main issues regarding datasets for 3D HPE. First, whether relying on manual labeling or motion capture devices, the production cost of 3D HPE datasets is very high, and the scarcity of datasets has become the primary problem that hinders the development of 3D HPE. Second, since most current 3D HPE datasets are created using motion capture devices, which can only capture the motions of people within a fixed range in specific environments, datasets for outdoor scenes are relatively scarce. Third, due to the limitations of motion capture devices, most of the current 3D HPE datasets are of daily behavioral actions with small amplitudes of limb movements and low speed, and there is a lack of recording of large amplitudes of limb movements such as dancing, sports, and abnormal behaviors. Fourth, there is a generalization issue between different datasets. For example, networks trained solely on datasets collected in controlled laboratories might struggle to accurately detect human motions in outdoor environments. Additionally, the domain gap between synthesized and real-world datasets often causes performance degradation when models trained on synthetic data are applied to real-world pose estimation tasks.

To address the scarcity of datasets, Pavllo et al. [80] adopted a semi-supervised training strategy that combines labeled data and expanded unlabeled data for joint training to further improve model performance. Yang et al. [117] proposed an unsupervised 3D HPE method based on mask information, leveraging easily obtainable human masks as supervision signals to eliminate the reliance on supervised post-processing in traditional approaches. The method designs skeleton masks and body shape masks, progressively refining the 3D pose from coarse to fine, and addresses the left–right symmetry issue through multi-view geometric constraints. Du et al. [118] proposed an iterative learning approach that simultaneously trains data augmentation and HPE, utilizing estimated poses to guide the training of the augmentation module, and employing reinforcement learning-based evolutionary data augmentation to enhance the credibility of generated 3D human pose data. Recent advances in cross-modal supervision have further expanded data-efficient learning paradigms. Ji et al. [119] introduced HPOF, which leverages optical flow as weak supervision by aligning projected 3D poses with frame-to-frame motion cues. Marcard et al. [108] fused IMU and video data in a joint optimization framework to generate accurate 3D annotations for monocular videos. Zhao et al. [120] used IMU data as auxiliary supervision during training to improve monocular 3D pose estimation under occlusion, demonstrating that limited IMU signals can guide robust vision-based predictions.

To mitigate the lack of datasets for outdoor scenes, Rogez et al. [121] proposed an image-based synthesis engine that can artificially generate outdoor datasets with 3D annotations by combining real image data with 2D annotations. Mehta et al. [78] utilized the character segmentation masks available in MPII [31] to synthesize multi-person 3D pose datasets with indoor and outdoor scenes at a large scale, containing complex multi-person interactions and occluded real images. Wang et al. [122] presented FreeMan, the first large-scale multi-view dataset for 3D pose estimation captured with eight smartphones in diverse real-world scenarios. The dataset includes 11M frames from 8000 sequences of 40 subjects across 10 scenarios with varying lighting conditions.

In tackling the scarcity of large-scale limb motion datasets, Zeng et al. [115] argued that local poses in rare poses can be obtained from other datasets and designed a network structure, SRNet, to split and reorganize the human body into local joint groups and recombine them to improve the accuracy of rare pose estimation.

Generalization across datasets remains a critical challenge. Peng et al. [123] proposed a novel framework that incorporates two pose augmentors. The weak augmentor simulates target domains similar to the source domain, while the strong augmentor emulates target domains that deviate significantly from the source distributions. The framework also leverages meta-optimization to simulate domain shifts during the optimization process of the pose estimator, thereby enhancing its generalization ability. Gong et al. [124] argued that the poor generalization of synthetic datasets is because of limited diversity in poses, body sizes, and camera viewpoints in the training data. They proposed an online data augmentation framework, PoseAug, which adjusts poses, body sizes, and camera viewpoints through differentiable operations, and the estimation error can be used as feedback to generate more varied poses online. Doersch et al. [125] found that motion information extracted from video sequences can help neural networks learn 3D HPE from synthesized images, and adding motion information to the 3D pose estimation model enhances its accuracy on actual datasets.

2. Depth Ambiguity

**Figure 13:**
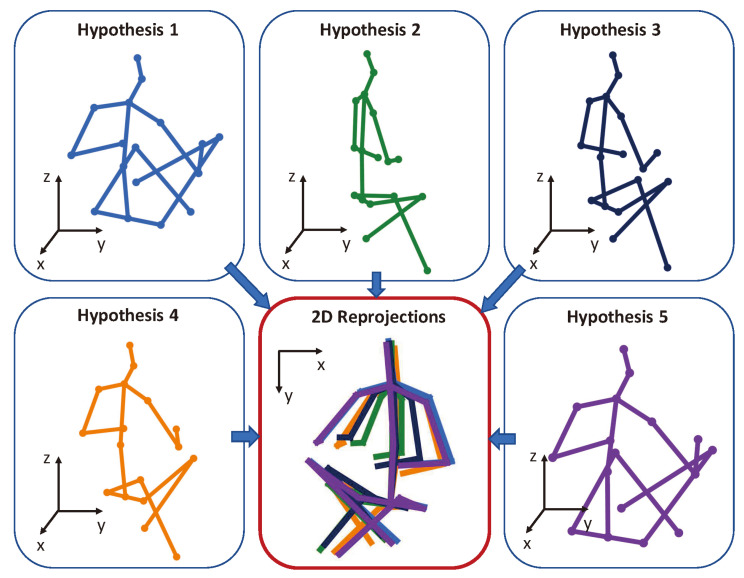
An example of depth ambiguity. Even with similar 2D projections, these pose hypotheses differ significantly in 3D. Part of the figure is from [113].

The essence of 3D HPE is to recover 3D poses from 2D information, essentially reconstructing high-dimensional data from low-dimensional inputs. Monocular RGB camera-based 3D HPE faces a severe challenge in depth ambiguity, as a single 2D image can correspond to multiple plausible 3D poses, as illustrated in Figure 13. This challenge is further intensified by the absence of direct depth information, unlike multi-camera systems [1] that infer depth through triangulation or depth sensors [3,4] that provide explicit depth measurements.

Three-dimensional HPE from monocular images is a highly ill-posed problem due to depth ambiguity. To address this problem, some methods [113,126,127] generate multiple potential 3D pose hypotheses from a single 2D image. The concept of generating multiple 3D pose hypotheses was initially proposed by Jahangiri et al. [127], who proposed a generative model to predict multiple 3D poses by uniformly sampling from learned occupancy matrices and incorporating constraints such as joint angles, and the generated results cover the full variability of the human 3D pose. Earlier approaches [113,126,127] have focused on producing 3D pose hypotheses, with little attention paid to how to aggregate these into a more precise 3D prediction. Shan et al. [61] aggregated multiple hypotheses by comparing them at the joint level with 2D keypoints, selecting the best matches to create the final 3D pose. Multi-hypothesis methods have the advantage of providing multiple possible solutions when faced with an ill-posed problem. However, generating multiple pose hypotheses increases computational costs and requires additional post-processing steps to select the best pose. Other methods [48,114] utilize GCNs to solve the deep ambiguity, where GCNs are able to capture global and local relational information from the connections between keypoints and the graph structure. By utilizing this information, GCNs can make more accurate estimations of keypoints in cases of deep ambiguity.

The root cause of the depth ambiguity problem lies in the limitations of monocular vision. A monocular RGB camera can only capture 2D RGB images and lacks direct depth information. Using depth cameras or depth images can effectively mitigate depth ambiguity by providing explicit spatial information, enabling more accurate and robust 3D human pose estimation. Karagoz et al. [3] proposed Dense Depth Alignment (DDA), leveraging dense depth maps as auxiliary supervision to improve 3D human pose and shape estimation by aligning estimated depth information with pose predictions, effectively addressing depth ambiguity. Carraro et al. [4] proposed a real-time, markerless 3D human pose estimation system using RGB-D cameras and multi-view fusion, enabling robust multi-person tracking while effectively addressing depth ambiguity by leveraging depth information and multi-perspective data integration.

3. Occlusion

**Figure 14:**
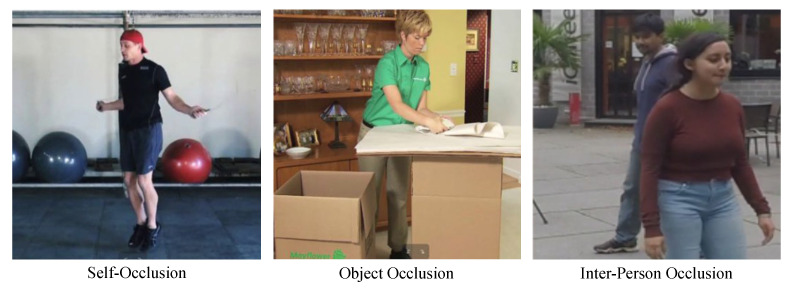
Illustration of human body occlusions including self-occlusion, object occlusion, and inter-person occlusion.

Human body occlusion means that some areas of the human body are occluded during image capture. Human body occlusion can be divided into three categories according to the source of occlusion: self-occlusion, object occlusion, and inter-person occlusion, as shown in Figure 14. The inability to include a complete human body in the input image creates great difficulties in the estimation of 3D human joints. First, monocular RGB models struggle to detect and infer occluded keypoints, as the absence of visual cues leads to incorrect predictions. In contrast, multi-camera systems [2] can leverage different viewpoints to reconstruct occluded joints, while depth sensors [5,6] provide explicit geometric information to mitigate occlusion effects. Secondly, since the joints of the human body are interrelated, the loss of certain keypoints similarly affects the predictions of unobscured keypoints. Therefore, it is imperative not only for algorithms to accurately estimate unobscured keypoints but also to possess the capability to extract more favorable features and make reasonable predictions for occluded keypoints.

To address the issue of human occlusion, some studies enhance model robustness and generalization by simulating occlusion conditions during training. Cheng et al. [128] randomly masked keypoint heatmaps to simulate three types of occlusion—frame-level, point-level, and region-level—allowing the model to learn how to infer missing keypoints. Shan et al. [129] randomly masked keypoints in both spatial and temporal domains during pre-training and used a denoising autoencoder to restore the original pose, improving occlusion robustness. Ghafoor et al. [130] utilized an occlusion guidance matrix derived from a 2D pose estimation network to assess the reliability of detected joints, assigned each joint a confidence score, and incorporated this binary occlusion guidance array as additional input to help the model differentiate between reliable and unreliable joint predictions.

Other studies argue that instead of relying on potentially incorrect full 2D keypoints, models should directly learn to recover 3D poses from incomplete but reliable 2D keypoints. Cheng et al. [131] used keypoint confidence heatmaps and optical flow consistency constraints to filter out unreliable occluded keypoints. Benzine et al. [69] proposed PandaNet, an anchor-based architecture that removes ambiguous anchors to address occlusion issues.

Self-supervised learning enhances robustness by simulating occlusions through data augmentation and generation, while leveraging uncertainty modeling to identify and adjust predictions in occluded regions for improved accuracy. Kundu et al. [132] introduced a self-supervised 3D human pose estimation framework that disentangles pose, camera parameters, and foreground appearance, leveraging part-guided novel image synthesis to enhance accuracy and robustness in complex scenes and occlusions. Two years later, they further developed MRP-Net [133], a self-supervised uncertainty-aware adaptation framework for 3D human pose estimation, which quantifies uncertainty at both pose and joint levels to refine uncertain predictions, enhancing reliability in handling occlusions and noise.

Additionally, some approaches leverage temporal information to handle occlusions, as occluded body parts in one frame may become visible in the next. Cheng et al. [134] introduce Velocity-TCN, which takes person-centered 3D poses and past velocity information as inputs to predict current actions. By analyzing motion cues from previous frames, the model estimates the current pose more robustly in occluded scenarios.

4. Insufficient Motion Smoothness and Motion Distortion

Between consecutive frames in a video, human body poses and motions are spatial–temporal correlated. However, traditional 3D HPE methods often process each frame independently, neglecting the utilization of temporal and spatial information. This disregard for temporal coherence can result in non-smooth pose estimation and may appear to be discontinuous or unnatural at motion transitions. Additionally, rapid or complex motions may lead to estimation results that exceed the normal range or shape of the human body.

To address the problem of insufficient motion smoothing, the temporal information in the video sequences can be utilized to enhance the spatial–temporal continuity and correlation of the pose estimation. Zheng et al. [9] proposed PoseFormer, which achieves performance improvements through a spatial transformer for local joint relationships and a temporal transformer for global frame dependencies. However, PoseFormer overlooks joint motion variations, resulting in insufficient learning of spatial–temporal correlations. Additionally, its larger temporal transformer dimension limits the use of longer sequences. Zhang et al. [89] proposed MixSTE to address the shortcomings of PoseFormer. By alternately employing temporal and spatial transformer to obtain spatial–temporal feature encoding, the model can better capture the global sequence coherence.

Additional constraints, such as bone length consistency, kinematic constraints, etc., can be introduced to address the motion distortion problem. Xu et al. [135] explicitly incorporated kinematic regularization into the depth model, achieving more accurate estimations from noisy 2D joint inputs. Chen et al. [116] followed the principle of bone length consistency over time for the human skeleton and decomposed the task into bone orientation prediction and bone length prediction. Shi et al. [136] proposed MotioNet, which incorporates embedded kinematic priors. In contrast to traditional methods [137], MotioNet avoids model-based assumptions and does not rely on inverse kinematics (IK) for joint inference. Instead, it directly infers joint rotations from training data.

**Table 9:** Performance comparisons of various methods under different challenges.

Challenge	Reference	Input	Frames	Network	Dataset	EP ^1^	Score
Scarcity of datasets	[80]	Video	243	TCN	Human3.6M	MPJPE	46.8
	[115]	Video	243	CNN	Human3.6M	MPJPE	44.8
	[124]	Image	-	CNN	Human3.6M	MPJPE	50.2
	[118]	Image	-	CNN	Human3.6M	MPJPE	47.0
Depth ambiguity	[126]	Image	-	Real-NVP	Human3.6M	MPJPE	44.3
	[48]	Image	-	GCN	Human3.6M	MPJPE	52.7
	[114]	Video	7	GCN	Human3.6M	MPJPE	48.8
	[61]	Video	243	Transformer	Human3.6M	MPJPE	40.0
Occlusion	[128]	Video	64	TCN	Human3.6M	MPJPE	41.2
	[129]	Video	243	Transformer	Human3.6M	MPJPE	42.1
	[131]	Video	128	TCN	Human3.6M	MPJPE	44.1
	[69]	Image	-	CNN	JTA [138]	3DPCK	83.2
	[134]	Video	243	GCN+TCN	3DPW	MPJPE	64.2
Motion smoothness	[9]	Video	81	Transformer	Human3.6M	MPJPE	44.3
	[89]	Video	243	Transformer	Human3.6M	MPJPE	40.9
Kinematic distortion	[116]	Video	243	CNN	Human3.6M	MPJPE	44.1
	[136]	Video	-	CNN	Human3.6M	MPJPE	54.59

^1^ EP indicates evaluation protocol.

## 7. Applications

**Figure 15:**
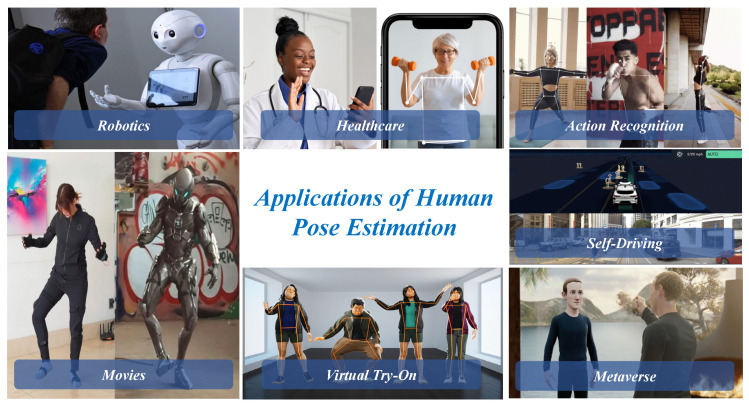
Examples of HPE application.

Three-dimensional HPE stands as a foundational computer vision task with significant relevance across various domains, including action recognition, metaverse, healthcare, self-driving, and many others, as shown in Figure 15.

1. Action Recognition

Action recognition is one of the downstream tasks of HPE and is widely applied in video surveillance for behavior analysis, anomaly detection, and security monitoring. HPE is more inclined towards the structured representation of the human body, obtaining only the pose parameters, which is a typical measurement task; while the goal of action recognition is to semantically understand human actions, which belongs to high-level classification tasks. Typically, information fusion of multiple measured parameters is required for action recognition, so the step of HPE is usually added in the middle of the network to classify the actions in combination with other information. Zhou et al. [139] proposed relative position embedding based on graph distances, which successfully integrates the skeletal structural information into transformer and applies hypergraph representations to skeleton-based action recognition.

By tracking human movements, action recognition can detect suspicious activities, such as violence and trespassing, enhancing public safety and automated surveillance systems. However, integrating HPE into surveillance raises ethical concerns regarding privacy, as continuous monitoring of individuals can lead to potential misuse or unauthorized data collection. To address this, researchers have explored privacy-preserving techniques, such as on-device processing [140], anonymization of human pose data [141,142], and differential privacy methods [143], ensuring that surveillance systems operate within ethical boundaries while maintaining their effectiveness.

2. Self-Driving

Three-dimensional HPE provides a detailed understanding of pedestrians and other road users in a traffic scenario. This helps self-driving systems identify and track pedestrians more accurately and predict their behavior for traffic safety. Huo et al. [144] proposed a 3D skeleton-aware driver behavior recognition framework that integrates a temporal transformer and spatial GCN for 3D pose estimation, along with a lightweight multi-stream CNN network for efficient and accurate behavior recognition based on 3D skeleton features, aiming to enhance in-cabin driver monitoring and safety in autonomous driving systems.

3. Virtual Try-On

With the development of e-commerce, static images of clothing no longer meet the needs of customers. By employing 3D HPE technology, virtual try-on and makeup functionalities can be achieved. Customers can upload their own photos or use a camera to capture their images and try on different clothing, shoes, accessories, or cosmetics in a virtual environment to better understand their appearance and fit. Patel et al. [145] proposed TailorNet, which predicts 3D garment deformations based on the three factors of human posture, shape, and style while preserving wrinkle details.

4. Movies

The creation of lifelike digital characters heavily relies on motion capture technology, which is often expensive and complex to set up. Affordable and precise motion capture systems can significantly contribute to the advancement of the digital entertainment industry. Three-dimensional HPE offers realistic posture information while reducing the reliance on expensive professional equipment. Liu et al. [146] introduced the PoseTween interactive system, allowing novice users to effortlessly add vibrant virtual objects whose movements interact with the moving subjects in the input video.

5. Metaverse

In the Metaverse, 3D HPE forms the foundation for creating realistic and responsive digital avatars. It allows users’ real-world movements to be accurately mapped to their virtual counterparts, enhancing immersion and interactivity in applications like virtual meetings, gaming, and training simulations. Yang et al. [147] introduced a camera-free human pose estimation framework that leverages ubiquitous WiFi signals, aiming to provide a privacy-friendly and robust solution for avatar simulation in the metaverse. Jiang et al. [148] introduced a method for full-body pose estimation using only head and hand motion data, addressing the limitations of body tracking in MR systems. Zhang et al. [149] proposed a system that transforms tennis match videos into controllable video sprites mimicking professional players.

6. Healthcare

With 3D HPE, medical professionals can monitor patients’ posture and motion to provide real-time feedback and evaluation of rehabilitation training. This is valuable for rehabilitation therapy, the prevention of sports injuries, etc. Lu et al. [150] designed a posture-based estimation system to assess motor severity in Parkinson’s disease.

7. Robotics

Three-dimensional HPE enables robots to accurately perceive human movements in real time, facilitating safer and more intuitive human–robot interactions. Additionally, it facilitates robotic imitation learning, allowing robots to observe and replicate human actions for improved efficiency in tasks like warehouse logistics. Amorim et al. [151] introduced a robust HPE method for cooperative robotics, integrating vision and IMU data to enhance safety and efficiency in industrial human–robot collaboration. Zimmermann et al. [152] proposed an RGB-D-based 3D HPE method that enables markerless learning from demonstration, allowing a PR2 robot to imitate human manipulation tasks for improved task learning and interaction.

## 8. Conclusions and Future Direction

This survey provides a comprehensive review of recent advances in monocular 3D human pose estimation, driven by the rapid evolution of deep learning techniques. It systematically categorizes existing approaches by their input modalities and architectural designs, while highlighting emerging paradigms such as SSMs and diffusion-based methods. By synthesizing the strengths, limitations, and innovations across the field, this survey serves as a valuable resource for researchers aiming to understand current trends and explore new avenues in 3D HPE. Furthermore, it reflects on existing challenges and discusses representative strategies proposed in recent studies as potential solutions. Building upon the analysis of current limitations, several promising research directions are outlined to support future advancements in monocular 3D HPE.

Building on these insights, several promising research avenues merit further exploration. From the perspective of data-efficient learning, existing 3D human pose estimation methods still heavily depend on large-scale manually annotated datasets, which are costly and time-consuming to obtain. Reducing the need for annotated data is an important trend. Future research may explore more self-supervised learning and unsupervised learning methods to reduce the cost of data annotation while improving the performance of the model.

In terms of cross-domain generalization, current 3D HPE models often struggle to generalize across different datasets due to variations in viewpoints, appearance, motion patterns, and environments. Future work could address the generalization gap by learning domain-invariant features, which guides the network to ignore domain-specific features and extract representations less sensitive to data distribution shifts. In addition, meta-learning can simulate domain shifts by dividing training data into “pseudo-domains”, improving generalization to unseen environments. Data augmentation methods can also enhance robustness by introducing more diverse visual appearances. These advancements will be essential for deploying pose estimation systems in real-world scenarios involving outdoor environments, diverse human physiques and clothing styles, different lighting conditions, and varying camera viewpoints.

Real-time performance remains a critical requirement for applications such as virtual reality, human–computer interaction, and motion capture. Future research will focus on how to improve the inference speed of models while maintaining accuracy. To achieve this, several strategies can be explored, including model compression techniques such as pruning and quantization and the design of lightweight network architectures tailored for low-latency inference. In addition, leveraging temporal redundancy in video sequences through frame skipping or motion priors may further reduce computational load without compromising prediction quality.

Recent advances in LLMs [153] have introduced new opportunities for enhancing 3D HPE. With their strong capacity for modeling semantic context, such as human actions, scene layouts, and interactions, LLMs can provide valuable prior knowledge to guide 3D pose estimation, particularly in complex or ambiguous scenarios. Future research may explore the integration of LLMs into 3D HPE frameworks to provide high-level semantic cues or auxiliary supervisory signals. In addition, LLMs can be employed to generate synthetic annotations, thereby improving label efficiency.

NeRF-based techniques offer significant potential for improving 3D HPE by capturing fine-grained spatial details from limited visual observations. Their ability to infer dense 3D structures from sparse viewpoints enables more accurate and robust pose estimation, particularly in scenarios involving occlusion or limited training data. Future research may explore how to effectively integrate NeRF into 3D HPE frameworks to enhance performance under such challenging conditions.

Integrating physics-based constraints into deep learning models can improve the realism and consistency of human pose estimation. By leveraging physical laws such as kinematics, dynamics, and biomechanical constraints, future research can develop models that generate more physically plausible poses, especially in scenarios with occlusions or ambiguous observations. Additionally, physics-informed models can enhance generalization to real-world environments by reducing reliance on large-scale annotated datasets.

## Figures and Tables

**Table 1:** Summary of related surveys on human pose estimation.

Survey	Year	Journal	Content
Holte et al. [10]	2012	*JSTSP*	Discussed multi-view HPE and activity recognition.
Chen et al. [11]	2013	*PRL*	Reviewed human motion analysis using depth imagery, covering sensor technologies, datasets, and recognition methods.
Perez-Sala et al. [12]	2014	*Sensors*	Reviewed model-based approaches for 2D and 3D human pose recovery, focusing on appearance, viewpoint, spatial relations, temporal consistence, and behavior.
Gong et al. [16]	2016	*Sensors*	Surveys monocular HPE, covering both traditional and deep learning-based methods.
Munea et al. [17]	2020	*IEEE Access*	Classified pose estimation by the number of people (single vs. multi-person), and briefly discussed significant papers for both cases.
Chen et al. [15]	2020	*CVIU*	Reviewed deep learning-based monocular HPE, categorizing methods into 2D and 3D approaches, and discussing datasets, evaluation metrics, and future directions.
Wang et al. [18]	2021	*CVIU*	Reviewed deep learning-based 3D HPE methods, categorizing them into monocular, multi-view, and sequential approaches, and discussed datasets, evaluation metrics, and future directions.
Liu et al. [19]	2022	*CSUR*	Provided a comprehensive 2D-to-3D overview of monocular HPE with deep learning, covering skeleton- and model-based methods and future directions.
Zheng et al. [14]	2023	*CSUR*	Reviewed deep learning-based methods for 2D and 3D human pose estimation, summarizing key approaches, benchmark datasets, and evaluation metrics.
Neupane et al. [20]	2024	*AIR*	Surveyed deep 3D HPE paradigms, explored alternative learning and data augmentation, categorized challenges, and proposed future directions.

**Table 2:** Comparison of the kinematic model and the volumetric model.

Feature	Kinematic Model	Volumetric Model
Construction method	Keypoints	Mesh
Occlusion handling	Less robust	More robust
Computational complexity	Low	High
Model detail	Low detail	High detail
Application scenario	Real time	Detail-oriented

**Table 3:** Comparison of end-to-end estimation and 2D-to-3D lifting methods.

Category	Type	References	Advantages	Limitations
End-to-end	CNN-based	[29,30]	Global image analysis, simple pipeline	High computational cost, environmental sensitivity
	Transfer learning	[31,32]		
	Capsule network	[34,35]		
2D to 3D	CNN-based	[40,41,42,43,44]	2D geometric relationships	Dependence on 2D keypoint accuracy
	GCN-based	[45,46,47,48,49,50,51]		
	Diffusion-based	[59,60,61,62,63,64,65]		

**Table 4:** Comparison of top-down and bottom-up approaches.

Category	References	Procedure	Region	Computational Complexity	Scale Sensitivity
Top-down	[68,69,70,71,72]	(1) Human detection (2) Estimate 3D pose	Cropped RoI	Grows with people	Low
Bottom-up	[74,75,76,77,78]	(1) Joint detection; (2) Joint clustering	Whole image	Linear complexity	High

**Table 5:** Comparative analysis of architectural characteristics for video-based 3D human pose estimation.

Architecture	RNN and LSTM	TCN	Transformer	Mamba
Reference	[81,82,83]	[80,84,85]	[9,79,89,91,92,93,94,96,99]	[60,103]
Parameter Quantity	Low	Moderate	High	Moderate
Parallel Processing	Low	High	High	High
Model Flexibility	Fixed	Moderate	Adaptable	Adaptable
Long-Term Dependency	Limited	Moderate	Strong	Strong
Gradient Stability	Unstable	Stable	Stable	Stable
Computation Cost	O(N)	O(N)	$O(N^{2})$	O(N)

**Table 6:** Quantitative performance benchmarking of video-based 3D human pose estimation architectures.

Architecture	Reference	2D Detector	Frames	Params (M)	FLOPs (M)	FPS	MPJPE
RNN	[81]	CNN	-	-	-	-	80.15
LSTM	[82]	CNN	-	-	-	-	55.8
	[83]	CPM [104]	-	16.95	33.88	-	58.3
TCN	[80]	CPN [105]	243	16.95	33.87	863	46.8
	[84]	CPN	243	11.25	-	-	45.1
	[85]	CPN	243	-	-	-	44.3
Transformer	[9]	CPN	81	9.6	1358	269	44.3
	[79]	CPN	243	4.23	1372	108	44.0
	[89]	CPN	243	33.7	645	-	40.9
	[93]	HR-Net [75]	96	3.7	-	-	42.6
	[90]	CPN	243	24.72	4812	-	43.2
Mamba	[101]	Hourglass [39]	243	6.7	-	-	38.1
	[102]	Hourglass	243	14.42	40.58	-	37.5

**Table 7:** Datasets commonly used in 3D HPE.

Datasets	Year	Annotation Type	Joint	Environment	Size	EP ^*a*^	MP ^*b*^	MV ^*c*^	Key Features
**Real Dataset**									
HumanEva	2010	3D joints	15	Indoor	40k frames	MPJPE	✕	✓	6 subjects, 7 actions
Human3.6M	2014	3D joints, mesh	17	Indoor	3.6M frames	MPJPE	✕	✓	11 subjects, 17 actions, bounding box, depth data
CMU Panoptic	2016	3D joints	15	Indoor	1.5M frames	3DPCK	✓	✓	8 subjects, depth data
MPI-INF-3DHP	2017	3D joints	15	Indoor, outdoor	1.3M frames	3DPCK	✕	✓	8 subjects, 8 actions, various scenarios
TotalCapture	2017	3D joints	26	Indoor	1.9M frames	MPJPE	✕	✓	5 subjects, 5 actions
3DPW	2018	3D joints, mesh	24	Indoor, outdoor	51k frames	MPJPE	✓	✕	7 subjects, 18 clothing styles
**Synthetic Dataset**									
NBA2K	2020	3D joints, mesh	35	Indoor	27k frames	MPJPE	✕	✕	27 subjects, 27k poses, basketball-specific
GTA-IM	2020	3D joints, mesh	98	Indoor	1M frames	MPJPE	✕	✕	50 subjects, RGB-D images
Occlusion-Person	2020	3D joints	15	Indoor	73k frames	MPJPE	✕	✓	13 subjects, RGB-D images, occlusion-focused

^*a*^ EP indicates evaluation protocol. ^*b*^ ✓ indicates including both single-person and multi-person scenarios; ✕ indicates including only single-person scenarios. ^*c*^ ✓ indicates including both single-view and multi-view scenarios; ✕ indicates including only single-view scenarios.

**Table 8:** Performance of 3D HPE methods on Human3.6M dataset in recent years (Protocol 1).

Approach	Reference	2D Detector	Input	MPJPE															
				**Direct**	**Disc.**	**Eat**	**Greet**	**Phone**	**Photo**	**Pose**	**Purch.**	**Sit**	**SitD.**	**Smoke**	**Wait**	**WalkD.**	**Walk**	**WalkT.**	**Avg**
End-to-end	[34]	-	Image	73.33	83.45	85.33	79.08	89.99	109.95	76.08	73.61	104.12	136.27	87.59	79.19	87.13	66.31	76.88	87.22
	[35]	-	Image	70.16	76.67	78.41	76.87	87.99	109.49	72.23	73.12	108.84	149.53	87.29	75.14	87.7	65.38	75.76	86.17
2D to 3D	[80]	CPN	Video	45.2	46.7	43.3	45.6	48.1	55.1	44.6	44.3	57.3	65.8	47.1	44	49	32.8	33.9	46.8
	[113]	Hourglass	Image	43.8	48.6	49.1	49.8	57.6	61.5	45.9	48.3	62	73.4	54.8	50.6	56	43.4	45.5	52.7
	[114]	CPN	Video	44.6	47.4	45.6	48.8	50.8	59	47.2	43.9	57.9	61.9	49.7	46.6	51.3	37.1	39.4	48.8
	[115]	CPN	Video	46.6	47.1	43.9	41.6	45.8	49.6	46.5	40	53.4	61.1	46.1	42.6	43.1	31.5	32.6	44.8
	[116]	CPN	Video	41.4	43.5	40.1	42.9	46.6	51.9	41.7	42.3	53.9	60.2	45.4	41.7	46	31.5	32.7	44.1
	[47]	CPN	Image	45.4	49.2	45.7	49.4	50.4	58.2	47.9	46	57.5	63	49.7	46.6	52.2	38.9	40.8	49.4
	[9]	CPN	Video	41.5	44.8	39.8	42.5	46.5	51.6	42.1	42	53.3	60.7	45.5	43.3	46.1	31.8	32.2	44.3
	[79]	CPN	Video	40.3	43.3	40.2	42.3	45.6	52.3	41.8	40.5	55.9	60.6	44.2	43	44.2	30	30.2	43.7
	[89]	CPN	Video	37.6	40.9	37.3	39.7	42.3	49.9	40.1	39.8	51.7	55	42.1	39.8	41	27.9	27.9	40.9
	[93]	HR-Net	Video	34.7	41.7	36	38.4	41.1	45.3	39.6	37.4	49	63.1	39.8	38.9	40.2	29.3	29.1	40.3
	[62]	Hourglass	Image	-	-	-	-	-	-	-	-	-	-	-	-	-	-	-	51.4
	[63]	CPN	Video	36.4	39.5	34.9	37.6	40.1	45.9	37.8	37.8	51.5	52.2	40.8	38.3	38.3	27.0	27.0	39.0
	[65]	CPN	Video	31.4	31.5	28.8	29.7	34.3	36.5	29.2	30.0	42.0	42.5	33.3	31.9	31.4	22.6	22.7	31.9

## Data Availability

Not applicable.
